# Chronic Subdural Hematoma Associated with Thrombocytopenia in a Patient with Human Immunodeficiency Virus Infection in Cameroon

**DOI:** 10.1155/2017/5395829

**Published:** 2017-01-10

**Authors:** Clovis Nkoke, Engelbert Bain Luchuo, Denis Teuwafeu, Ines Nepetsoun, Cyrille Nkouonlack

**Affiliations:** ^1^Faculty of Medicine and Biomedical Sciences, University of Yaounde 1 and Buea Regional Hospital, Yaounde, Cameroon; ^2^Center for Population Studies and Health Promotion, Yaounde, Cameroon; ^3^Buea Regional Hospital, Southwest Region, Cameroon

## Abstract

Hematological abnormalities including thrombocytopenia are common in patients living with HIV infection. Patients with HIV infection related thrombocytopenia present generally with only minor bleeding problems. But cases of subdural hematoma are very rare. A 61-year-old female with a history of HIV infection of 9 years' duration presented with a 3-month history of generalized headache associated with visual blurring and anterograde amnesia. There was no history of trauma or fever. She was treated empirically for cerebral toxoplasmosis for 6 weeks without any improvement of the symptoms. One week prior to admission, she developed weakness of the left side of the body. Clinical examination revealed left-sided hemiparesis. Computed tomography scan of the brain showed a 25 mm chronic right frontoparietotemporal subdural hematoma compressing the lateral ventricle with midline shift. There was no appreciable cerebral atrophy. A complete blood count showed leucopenia and thrombocytopenia at 92,000 cells/mm^3^. Her CD4-positive cell count was 48 cells/mm^3^ despite receiving combination antiretroviral therapy for 9 years. A complete blood count analysis suggestive of thrombocytopenia should raise suspicion of possibilities of noninfectious focal brain lesions like subdural hematoma amongst HIV infected patients presenting with nonspecific neurological symptoms. This will enable prompt diagnosis and allow early appropriate intervention.

## 1. Introduction

Human immunodeficiency virus (HIV) infection causes hematological abnormalities including thrombocytopenia [1, 2]. These hematological abnormalities have been reported as strong independent predictors of morbidity and mortality in individuals living with HIV infection [3]. They are most common during the advanced stage of the disease. Although the mechanism of these abnormalities still remains obscure, they may occur as a result of HIV infection itself, as sequel of HIV-related opportunistic infections or malignancies or as a consequence of drugs used for HIV infection treatment and associated conditions [1]. Thrombocytopenia may occur at any time during the course of HIV infection, but the incidence generally correlates with the degree of immunosuppression and is more prevalent in individuals with clinical AIDS [4].

HIV-associated thrombocytopenia is often asymptomatic but can manifest clinically as minor submucosal bleeding problems. Nevertheless, life-threatening hemorrhagic episodes such as subdural hematomas and subarachnoid hemorrhage are very rare. We report a case of subdural hematoma in a 61-year-old HIV infected patient associated with thrombocytopenia.

## 2. Case Presentation

**Figure 1:**
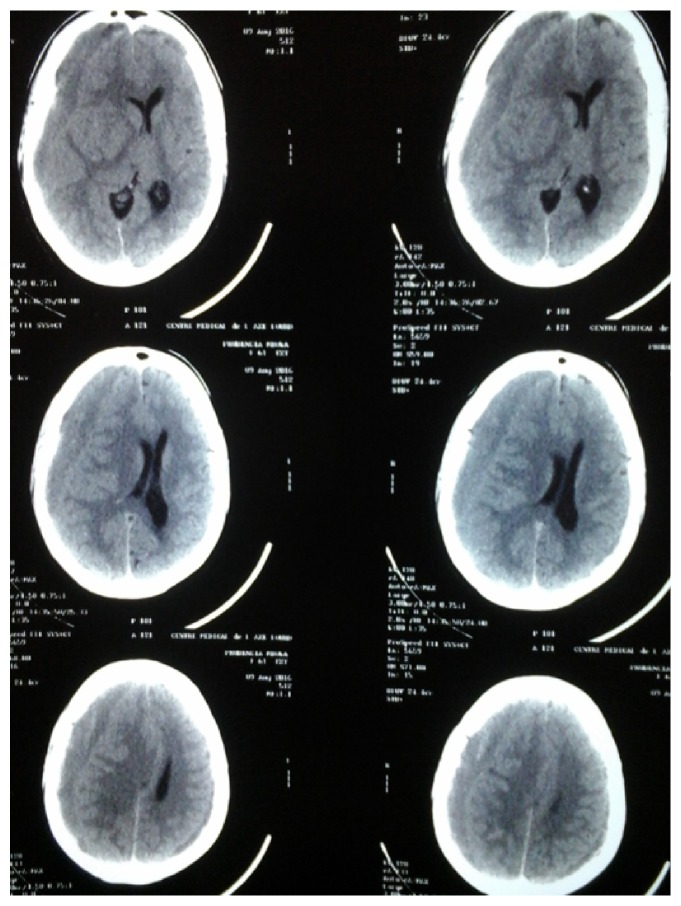
Computed tomography scan of the brain showing a right chronic subdural hematoma with midline shift.

A 61-year-old female patient with history of human immunodeficiency virus infection of 9 years' duration presented with a 3-month history of diffuse headache associated with visual blurring, anterograde amnesia. The headache was worse in the morning and at the end of the day. There was neither seizure nor focal neurologic deficits. She did not report any vomiting or fever. There was no history of trauma, hypertension, liver disease, or alcohol consumption. Her combined antiretroviral treatment regimen consisted of zidovudine, lamivudine, and nevirapine and she was compliant to treatment. She consulted the HIV treatment center for the headache where she was treated empirically for cerebral toxoplasmosis with cotrimoxazole for six weeks without any improvement of the headaches. One week prior to admission, she presented with limping and weakness of the left side of the body. There were no seizures. Clinical examination revealed a fully conscious patient with a left proportional hemiparesis. There was no sensory deficit. A computed tomography scan of the brain showed a 25 mm chronic right frontoparietotemporal subdural hematoma compressing the lateral ventricle with midline shift (Figure 1). There was no appreciable cerebral atrophy. The CD4 count was 48 cells/mm^3^; full blood count showed leucopenia with a total white blood cell count of 1600/mm^3^ with 992 neutrophils/mm^3^, a hemoglobin of 13 g/dL with macrocytosis (MCV = 115 fl), and thrombocytopenia with a platelet count of 92,000/mm^3^. The viral load was not determined. The hematoma was evacuated via a burr hole. The operation and recovery were uneventful. The patient did not receive any blood products perioperatively. The patient did not experience any bleeding problem during and after the surgery. The patient was discharged on day 7 after surgery.

## 3. Discussion

We have reported a case of chronic subdural hematoma associated with thrombocytopenia in a patient living with HIV infection with severe immunodepression in Cameroon. 

The most common cause of chronic subdural hematoma is trauma, in a predisposed brain. Most patients with chronic spontaneous subdural hematoma will remember some type of minor trauma [5]. In our patient, after a thorough interrogation, we could not evoke any history of trauma. AIDS related cerebral atrophy may predispose patients to the development of extracerebral collection. But the computed tomography scan in our patient did not show any appreciable cerebral atrophy that could have predisposed her to subdural bleeds. There was no history of hypertension, liver disease, or alcohol consumption in our patient which are all conditions that predispose to subdural bleeding. HIV-associated thrombocytopenia with possible associated dysfunctional platelets may have predisposed our patient to the development of subdural hematoma. She did not have any evidence of bleeding elsewhere.

A meta-analysis reviewing thrombocytopenia in HIV infected individuals before combined antiretroviral therapy concluded that the prevalence of HIV-associated thrombocytopenia is 5–30% [6]. In approximately 10% of the patients, it may be the first sign of AIDS [7]. Patients may present with thrombocytopenia at any time during the course of HIV infection, from asymptomatic infection to advanced AIDS. Thrombocytopenia is correlated with low CD4 cell count and older age as observed in our patient [8]. Although the pathogenesis has not been recognized, thrombocytopenia in HIV infected patients may arise through several mechanisms, including decreased platelet production, increased platelet destruction due to HIV-mimetic antiplatelet antibodies, and increased use of activated platelets [9]. Our patient was treated empirically with cotrimoxazole for cerebral toxoplasmosis when she presented with nonspecific neurologic symptoms. Though cotrimoxazole has been shown to directly affect thrombopoiesis, it may be difficult to determine its contribution to the thrombocytopenia in this patient. Thrombocytopenia is associated with increased morbidity and mortality, accelerated deterioration in CD4 counts, and accelerated progression to AIDS [10]. Thrombocytopenia predisposes to bleeding but the incidence of major bleeding is low in HIV-associated thrombocytopenia [11]. It is true that the risk of bleeding increases with the severity of the thrombocytopenia. Major bleeding is unusual unless the platelet count is less than 50 × 10^3^ per *μ*L. Risk factors for bleeding at higher platelet counts are structural lesions with loss of vascular integrity and dysfunctional platelets. We could not identify any other evident risk factors for the subdural hematoma except the thrombocytopenia. Associated dysfunctional platelets may have contributed to the subdural hematoma in our patient.

Spontaneous chronic subdural hematoma in HIV patients with normal platelets has been reported in the literature. It was postulated that these chronic subdural hematomas occurred as a complication of the coagulopathy in HIV infection particularly with low CD4 counts in retroviral infection, particularly with low CD4 counts [12]. HIV infection predisposes to both bleeding and thrombosis, and the mechanisms are believed to be multifactorial with some still to be elucidated ranging from blood vessel vasculitis to true blood coagulopathies.

Although thrombocytopenia is common in HIV infected patients, subdural hematoma is an uncommon presentation of this abnormality with few cases reported in the literature [13]. In their report, Raghurama Rao et al. reported a case of chronic subdural hematoma in HIV infected patient with thrombocytopenia [13]. Their patient was significantly younger than our patient.

Jokonya et al. reported spontaneous chronic subdural hematomas in human immunodeficiency virus-infected patients with normal platelet count and no appreciable brain atrophy. They hypothesized that dysfunctional platelets were the cause of the bleeding [12]. Combined antiretroviral therapy alone is often sufficient to correct thrombocytopenia and the development of recurring thrombocytopenia upon combined antiretroviral therapy discontinuation in some patients further strengthens the correlation [14]. Our patient despite being on combined antiretroviral therapy for many years had a very low CD4+ cell count and thrombocytopenia suggesting suboptimal therapy or adherence to therapy.

## 4. Conclusion

Thrombocytopenia is common in patients with HIV infection and presents only with minor submucosal bleeding. But life-threatening bleeding such as subdural hematoma is very rare and should be considered in HIV patients presenting with nonspecific neurologic symptoms. This will enable prompt diagnosis and allow early appropriate intervention.

## Abbreviations

HIV: Human immunodeficiency virus
AIDS: Acquired immunodeficiency syndrome.
